# Epithelial growth factor receptor expression influences 5-ALA induced glioblastoma fluorescence

**DOI:** 10.1007/s11060-017-2474-0

**Published:** 2017-05-12

**Authors:** Andrea O. Fontana, Deborah Piffaretti, Francesco Marchi, Floriana Burgio, Ana Bela Faia-Torres, Paolo Paganetti, Sandra Pinton, Uwe Pieles, Michael Reinert

**Affiliations:** 1 Laboratories of Biomedical Neurosciences (LBN), Department of Neurosurgery, Neurocenter of Southern Switzerland, Ente Ospedaliero Cantonale, Ticino, Switzerland; 2 FHNW, Muttenz, BL Switzerland; 3 Neurosurgery, Neurocenter of Southern Switzerland, Via Tesserete 46, 6903 Lugano, Switzerland

**Keywords:** Glioblastoma, 5-ALA, EGF, EGFR, EGFR overexpression, EGFRvIII, Heme Oxygenase-1, Tin(IV)-protoporphyrin, Gefitinib

## Abstract

The extent of 5-aminolevulinic acid (5-ALA) guided tumor resection has a determining impact in high-grade glioma and glioblastoma surgery. Yet the intensity of the 5-ALA induced fluorescence may vary within the tumor. We aimed to correlate 5-ALA induced fluorescence with the expression of epithelial growth factor receptor (EGFR) and its constitutively active version EGFRvIII in different glioblastoma (GBM) cell lines. To elucidate the role of EGFR in the metabolism of 5-ALA in GBM cell lines with variable EGFR expression status, we analyzed the activation of EGFR by its primary ligand EGF, and its downstream effect on Heme oxygenase-1 (HO-1), a key enzyme regulating the metabolism of Protoporphyrin IX (PpIX), the fluorescent metabolite of 5-ALA. Effects of direct pharmacological inhibition by Tin(IV)-Protoporphyrin (SnPP) or gene knockdown by small interfering RNA (siRNA) on HO-1 enzyme were analyzed in respect to 5-ALA induced fluorescence. Furthermore, inhibition of EGFR by Gefitinib was tested. A significant difference in 5-ALA induced fluorescence was obtained in U87MG (low EGFR expression) and LN229EGFR cells (EGFR overexpression) compared to BS153 (EGFR overexpression/EGFRvIII+). Treatment of U87MG and LN229EGFR cells with EGF significantly reduced cellular fluorescence, by promoting HO-1 transcription and expression in a concentration-dependent manner. This effect could be reversed by EGFR-specific siRNA treatment, which reduced protein expression of about 80% in U87MG. Remarkably, inhibition of HO-1 activity by SnPP or reduction of HO-1 protein levels by siHO-1 treatment restored fluorescence in all cell lines, independently of EGFR quantitative and qualitative expression. Gefitinib treatment was able to restore fluorescence after EGF stimulation in U87MG cells but not in BS153 cells, overexpressing EGFR/EGFRvIII. In GBM cell lines, 5-ALA induced fluorescence is variable and influenced by EGF-induced downstream activation of HO-1. HO-1 protein expression was identified as a negative regulator of 5-ALA induced fluorescence in GBM cells. We further propose that co-expression of EGFRvIII but not quantitative EGFR expression influence HO-1 activity and therefore cellular fluorescence.

**Electronic supplementary material:**

The online version of this article (doi:10.1007/s11060-017-2474-0) contains supplementary material, which is available to authorized users.

## Introduction

High-grade gliomas and especially glioblastoma (GBM) is the most common and progressive tumor of the central nervous system. Despite current multimodality treatments including maximal surgical resection, followed by a combination of radiotherapy and chemotherapy, the median survival is typically limited to 14 months after diagnosis [1, 2]. The extent of resection in patients with high-grade glioma and GBM can primarily impact overall survival, but it has to be carefully balanced with preserving the functional and neurological status of the patient in eloquent areas [3]. Over the last several years, 5-aminolevulinic acid (5-ALA) has been established as an intra-operative tool to augment the extent of resection in high-grade glioma and GBM surgery [4]. Clinically, the exogenous, oral administration of 5-ALA is usually performed 4h prior to surgery. 5-ALA is resorbed through the upper intestine into the blood, where it diffuses through the blood–brain barrier, which has been typically disrupted by infiltrative tumor cells [5–9]. During fluorescence assisted tumor resection a variance of the fluorescence intensity within GBM can be observed, especially at the infiltrating zone [4, 8, 10]. 5-ALA induced fluorescence may vary upon many factors such as cell density, proliferation index, mitochondrial mass and index and furthermore exogenous factors such as application or fading during surgery [5–8]. Schwake et al. have shown that in GBM cell lines continuous exposition of 5-ALA continuously increased 5-ALA induced fluorescence up to 24 h [11].

5-ALA is an indirect fluorophore and an early precursor molecule in the heme biosynthetic pathway, endogenously synthesized from the cells starting from glycine and succinyl-CoA.

Upon uptake into the cell, 5-ALA is metabolized by several enzymatic steps first within the cytoplasm and then into in the mitochondrial membrane to Protoporphyrin IX (PpIX), a compound capable of fluorescence when excited by appropriately filtered light. PpIX is further converted into heme, a non-fluorescent compound, by the enzyme Ferro-chelatase (FECH) [9]. Several studies have demonstrated that in general, tumor and highly proliferating tissues tend to exhibit higher level of PpIX than normal cells after 5-ALA incubation [12]. Indeed, multiple primary and metastatic CNS neoplasms notably take up preferentially exogenous 5-ALA and store it as PpIX, thus facilitating the neurosurgeon to accomplish an easier and more accurate tumor resection [13].

Although the PpIX synthesis does not solely result from the Porphobilinogendeaminase (PBDG) production and the FECH conversion, the balance does play an important role in the PpIX accumulation [9]. Other factors contributing to PpIX accumulation, specific to GBM, are reported from in vitro studies and include the NADPH imbalance and its effect on HO-1 [14]. Furthermore, Gandini et al. reported the higher frequency of HO-1 protein expression in human gliomas compared to non-malignant brain samples, justified by the HO-1 modulation of glioma cell proliferation. The effect is discussed to be in relation to the downstream effects of the products the PpIX metabolism [15].

The epithelial growth factor receptor (EGFR, also called ErbB-1), a member of the ErbB receptor family, is a 170-kDa glycosylated cell-surface receptor with tyrosine kinase activity [16]. EGFR has been thoroughly associated with several distinct steps in tumorigenesis, cell migration and angiogenesis. Upon binding of its natural ligand, the epidermal growth factor (EGFXX), the EGFR undergoes a conformational change which promotes dimerization, activation of the intracellular tyrosine kinase and autophosphorylation. This results in the activation of signal transduction molecules, including PI3 kinase, Akt and nuclear factor (NF-kβ) proteins, ultimately leading to tumor proliferation and progression, reduced apoptotic capacity, angiogenesis and the metastatic phenotype [17].

EGFR can exert oncogenic effects by different mechanisms, such as autocrine growth factor loops, amplification of the EGFR gene and deletions/mutations that render the receptor constitutively active [18, 19]. The most common mutation of the EGFR gene is a deletion of exons 2–7, producing an extracellularly truncated, constitutively-active EGFR, named EGFR variant III (EGFRvIII). Approximately 40% of GBM show EGFR gene amplification resulting in protein overexpression; among these ~50% express EGFRvIII [20, 21]. Remarkably, EGFRvIII expression is associated with concomitant (over) expression of the wild-type EGFR gene [22]. Hence, GBM could be subdivided into EGFR wild-type, EGFR overexpressing and EGFR/EGFRvIII co-expressing tumors.

All this considered, EGFR currently represents an attractive and promising target in high grade and GBM therapy. Yet intratumoral heterogeneity of EGFR/EGFRvIII expression in GBM may ultimately limit and challenge diagnostic and therapeutic utility in the clinical setting [23].

Expression of the *HO-1* gene (HMOX.1) is primarily regulated at the transcriptional level by activating transcription factors such as NF-kβ, AP-2, and the heat shock-responsive element (HSE) [24–26]. Several reports showed that EGF-induced NF-kB activation occurs through multiple EGFR-dependent signaling molecules, including PI3K, protein kinase C (PKC), and IKK signaling pathways [27].

**Fig. 1:**
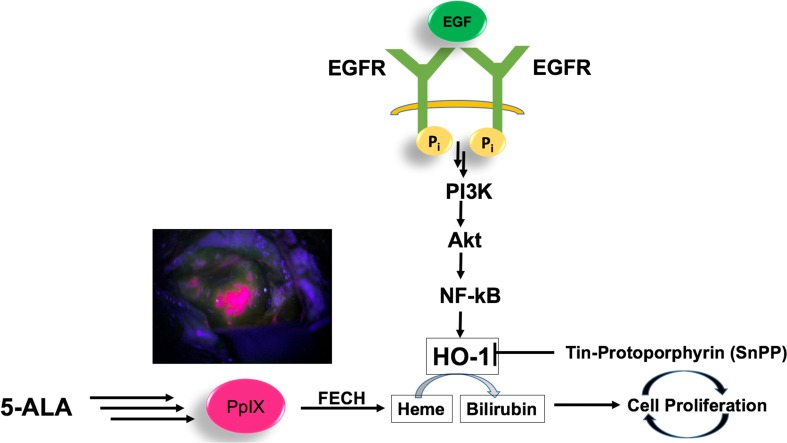
Representation of the 5-ALA metabolism as a function of the regulation of EGFR in GBM cells. EGF/EGFR signaling promotes HO-1 expression and activity in GBM cells through activation of the PI3K/AKT/NF-κB cascade, leading to cell proliferation. *5-ALA* 5-aminolevulinic acid, *EGF* epidermal growth factor, EGFR *epidermal growth factor receptor, HO-1* heme oxygenase-1, *PI3K* phosphatydil-inositol-3-kinase, *NF-kB* nuclear factor k-beta

We were particularly interested to understand how EGF regulates the induction of HO-1 protein expression in cancer cells. We hypothesized that in GBM cells, EGFR activation by its main ligand EGF may act as a *negative regulator* of 5-ALA-induced PpIX fluorescence, through induction of the enzyme HO-1 (Fig. 1).

Here, we use GBM cell lines with different EGFR expression levels to elucidate the molecular role of EGFR activation in 5-ALA-induced fluorescence.

## Materials and methods

### Cell lines

The human GBM cell line U87MG (Sigma-Aldrich, USA) was cultured in Dulbecco’s Modified Eagle Medium (DMEM, 61965-026, gibco^®^ Life technologies™, UK) GlutaMAX cell culture medium supplemented with 10% fetal bovine serum (FBS, 10270-106, gibco^®^ Life technologies™, UK), 1% non-essential amino acids (NEAA, 11140-035, gibco^®^ Life technologies™, UK), 1 mM sodium pyruvate and penicillin–streptomycin (100–100 μg/ml) (S8636, Sigma-Aldrich, USA). The human GBM cell line BS153 was kindly provided by the laboratories of Prof. Monika Hegi (Laboratory of Brain Tumor Biology and Genetics—University Hospital Lausanne, Switzerland) and was maintained in DMEM GlutaMAX, 10% FBS and 1% penicillin–streptomycin. BS153 is a GBM cell line immortalized first by Jones et al., which has retained amplification of the EGFR gene and expression of the EGFRvIII+ [28] LN229 cells overexpressing the EGFR gene (LN229EGFR) were generously provided by Proffesor Michael Weller (Department of Neurology, University Hospital Zurich, Switzerland) and maintained in DMEM GlutaMAX, 10% FBS and 1% penicillin–streptomycin, enriched with Hygromycin GOLD 60ug/ml.

As a control cell line, we used the immortalized astrocyte cell line IMA2.1, kindly provided by Dr. Stefan Schildknecht (University of Konstanz, Germany) [29], cultured in DMEM GlutaMAX, 10% FBS and penicillin–streptomycin. All cells were kept at 37 °C, 5% CO_2_ atmosphere, in static conditions.

### Drug treatment

5-ALA was obtained directly from the Hospital Pharmacy (Fagron DAC 2011, Germany) and freshly dissolved in distilled water. Cells were incubated for 18 h. EGF (E9644, Sigma-Aldrich, USA) was reconstituted in RNAase- and DNAase-free water and added to the cells for 18 h. The continuous exposition of cell lines to 5-ALA in comparison to short exposition times, has been previously reported to prevent fading of 5-ALA induced fluorescence for up to 24 h [11]. SnPP (CAS 14325-05-4, Santa Cruz Biotech, USA) was dissolved in DMSO and added to the cells 1 h prior to 5-ALA treatment. Gefitinib was ordered from Sigma–Aldrich (St. Louis, MO, USA), dissolved in DMSO to a final concentration of 10–20 µM and added to the cell culture 2 h prior to EGF treatment.

### Antibodies

The following antibodies were used:

Rabbit monoclonal anti-EGFR (ab52894, Abcam, Cambridge, UK); mouse monoclonal anti-EGFR (sc-120, Santa Cruz Biotechnology, USA); mouse monoclonal anti-EGFRvIII (L8A4, Absolute Antibodies, UK); rabbit polyclonal anti-EGFR (ab5652, Abcam, UK); mouse monoclonal anti-HO-1 (ab13248, Abcam, UK); mouse monoclonal anti-β-actin (AC-15, Sigma-Aldrich, USA); IRDye^®^ 800CW goat anti-mouse IgG (827-08364, LI-COR Biotechnology, Germany); IRDye^®^ 680RD goat anti-rabbit IgG (926-68071, LI-COR Biotechnology, Germany).

### siRNA transfection

Transfection of U87MG cells was performed 24 h before plating. siRNAs were used at a concentration of 25 nM (siEGFR) and 10 nM (siHO-1) with 1.7 μl/ml Lipofectamin RNAiMAX (18324-012, Invitrogen™ Thermo Fisher Scientific, USA). The following siRNA sequences were used: siCtrl: 5′-CGTACGCGGAATACTTCGAdTdT-3′; siEGFR and siHO-1: Dharmacon™ ON-TARGET plus SMARTpool against human EGFR (L-003114-00, GE Healthcare Europe GmbH, Switzerland) and human HMOX1 (L-006372-00, GE Healthcare Europe GmbH, Switzerland).

### Confocal microscopy

Cells were plated into Ibidi μ-slides VI0.4 (80606, Ibidi, Munich). The next day medium was substituted with FBS-free medium for at least additional 24 h prior to treatment with 1–10 ng/ml EGF. Following incubation with EGF, 1 mM 5-ALAwas added to the cell culture. At the desired time point, cells were washed twice with PBS (10010-015, Gibco^®^ Life technologies™, UK), fixed with 4% paraformaldehyde/PBS for 10 min, washed with PBS (4 × 5 min) and stored at 4 °C until staining. Cells were permeabilized for 5 min with 0.5% ice cold Triton-X-100/PBS, blocked for at least 20 min with 1% BSA, followed by 1 h incubation with the selected primary antibody, diluted in 1% BSA/PBS. After washing with 1% BSA/PBS (3 × 10 min), cells were incubated with the appropriate secondary antibody diluted 1:1000 (Alexa Fluor^®^ 488 and/or Alexa Fluor^®^ 594; Invitrogen), washed with 1% BSA/PBS (2 × 10 min) followed by PBS (1 × 10 min) and incubated with 1 μg/ml DAPI/methanol for 3 min and washed with PBS (3 × 10 min). Slides were examined with a Nikon ECLIPSE Ti-E confocal microscope and a Nikon C2 camera. Merged z-stacks (z = 0.49 µm) were analyzed with NIS-Elements AR software (Nikon). At least 50 cells/condition were analyzed.

### Flow cytometry analysis

To quantify the extent of PpIX biosynthesis induced by 5-ALA, we measured cellular fluorescence intensity in living cells using a flow cytometer (FC, AT01008, Navios™, Beckman Coulter), as previously described [30]. Cells were plated into 10 cm dishes and transfected with either mock or siHO-1 RNA. The next day, medium was substituted with FBS-free medium for at least 24 h prior to treatment with EGF. Following 18 h of EGF treatment, 5-ALA was added to the cell culture and at the desired time points, cells were harvested in the dark for FACS analysis. The auto-fluorescence intensity of PpIX, following 5-ALA administration, was measured with excitation at 488 nm and an emission peak of PpIX corresponding to 620 nm (605–635 nm) [31]. Auto-fluorescence signal of cells (fluorescence signal obtained in the absence of 5-ALA treatment) was also quantified and plotted as “untreated” in all the graphs shown in the “Results” section. The intensity of the fluorescence signal in the cell lines was quantified by computerized calculation (Kaluza Version 1.2, Beckman Coulter) by calculating the geometric means of the data distributions (G_mean_) from the signal plot. FACS parameters were always kept equal among all measurements.

To quantify the expression of EGFR and EGFRvIII in U87MG and in BS153, receptor density was measured by flow cytometry. In brief, cells were plated into 10 cm dishes and at the desired time points harvested and washed with ice cold PBS. Cells were then re-suspended and diluted to approximately 5 × 10^6^ cells/ml. Primary antibody in 3% BSA/PBS was added and cells were incubated for 1 h at 4 °C in the dark. After incubation, cells were centrifuged at 18,406×*g* for 5 min and fixed for 15 min with 100 µl of 4% paraformaldehyde (pH 7.4). Following fixation, cells were washed for three times by centrifugation (18,406×*g* for 5 min) and then incubated with the secondary antibody Alexa Fluor^®^ 488 (Invitrogen). After incubation cells were washed twice and then resuspended in FACS buffer (1% bovine serum albumin, 0.1 M EDTA in PBS) before analysis by FACS.

### Western blotting

At the indicated time points, cells were collected in PBS and centrifuged. The cell pellet was suspended in RIPA buffer and rested in ice for 30 min, followed by centrifugation at 21,130×*g* for 15 min. Cell extracts were collected, frozen in dry ice and stored at −80 °C until analysis. Equal protein amounts (30 µg) were run on a 10% polyacrylamide gel and blotted onto polyvinylidene difluoride (PVDF, 162-0177, Bio-Rad, USA) membranes by semi-dry electroblotting. Membranes were stained with Ponceau S to verify equal protein loading per lane. Membranes were blocked for 1 h with blocking buffer (Blocking solution (927-50000, LI-COR, Germany), TBS (200 mM Tris/Cl, 1.5 M NaCl, HCl, Milli-Q water), Milli-Q water). All antibodies were diluted in antibody dilution buffer (Blocking solution, TBS 0.1% Tween 20, Milli-Q water). Primary antibody detection was achieved through the use of the following secondary antibodies: IRDye^®^ 800CW goat anti-mouse IgG (827–08364, LI-COR Biotechnology, Germany); IRDye^®^ 680RD goat anti-rabbit IgG (926-68071, LI-COR Biotechnology, Germany).

Real-time PCR. To amplify human colon cancer cell HO-1 mRNA, specific primers were synthesized. The HO-1 primers used were: 5′-TAGAAGAGGCCAAGACTGCG-3′ (forward, Microsynth, Switzerland) and 5′-CAGAATCTTGCACTTTGTTGC-3′ (reverse, Microsynth, Switzerland). GADPH mRNA levels were used as an internal control. At the indicated time points, cells were harvested and RNA was extracted using TRI-REAGENT (Molecular Research Center, USA) according to the manufacturer’s instructions. For the reverse transcription reaction we used oligo-dT primers (GoScript™ Reverse Transcription System, A5000, Promega, Switzerland). The reaction was incubated at 42 °C for 15 min, then samples were heated at 95 °C for 5 min, followed by incubation at 0–5 °C for 5 min. Real-time RT-PCR was performed using SYBR^®^ Green Supermix (172-5271, SsoAdvanced™ Universal, Bio-Rad, USA) according to the manufacturer’s instructions. Briefly, equal amounts of each cDNA were PCR-amplified with Tag polymerase in at least 35 cycles consisting of 30 s at 95 °C, 30 s at 58 °C, and 1 min at 72 °C. At the end of the PCR, a melting curve analysis was performed by gradually increasing the temperature to 95 °C. Data acquisition was performed during the elongation step. After PCR completion, the SYBR Green fluorescent signal was transformed into a relative number of copies of target molecules. Differences in cDNA amounts were normalized as a function of the expression of the housekeeping gene GADPH.

### Statistical analysis

Data were presented as the mean ± standard deviation (SD) of at least three independent experiments. Statistical analysis was performed using Prism 5 GraphPad (version 7 for Windows, USA, http://www.graphpad.com). A Shapiro–Wilk test was used to determine the data normality. The results indicated that parametric tests should be used for the comparisons. The results were then tested for significance using the two-sided unpaired Student’s *t* test. Results were considered statistically significant when p < 0.0.5 (*).

## Results

### 5-ALA-derived fluorescence varies among GBM cell lines with different EGFR expression

**Fig. 2:**
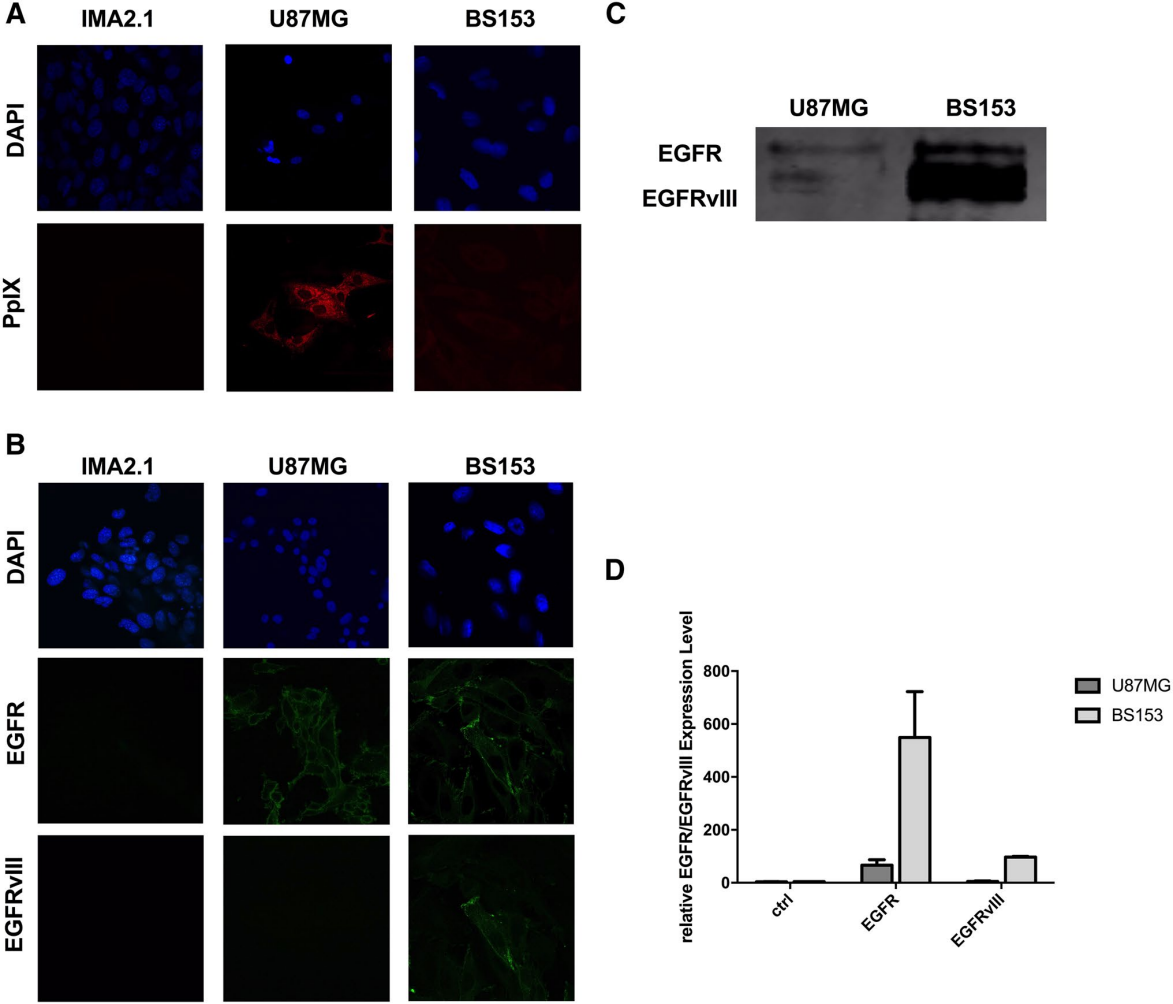
5-ALA fluorescence variability among GBM cells with different EGFR status. Protoporphyrin IX fluorescence after treatment with 5-ALA (**a**) among different GBM cell lines with variable EGFR/EGFRvIII expression status (**b**). EGFR and EGFRvIII protein levels as assessed by western blot (**c**) and flow cytometry (**d**, fluorescence relative to control conditions, i.e. no primary antibody, only with the secondary antibody)

Some clinical reports have shown that during 5-ALA induced fluorescence for guided tumor resection, some GBMs may exhibit areas, especially at the infiltrating zone, with weak to no fluorescence perceptible to the naked eye [4, 10]. We screened glial and GBM cells for their PpIX fluorescence intensity after treatment with 5-ALA (Fig. 2a). Positive fluorescence was found for both GBM cell lines U87MG and BS153, with significantly more intense PpIX fluorescence in U87MG when compared to BS153 cells. In contrast, the non-cancerous glial cell line IMA2.1 did not exhibit PpIX fluorescence. Analysis by immunofluorescence of U87MG cells confirmed the co-localization of PpIX with TOMM20, a known mitochondrial membrane marker (Fig. S1, supplementary material). Moreover, analysis by flow cytometry confirmed maximal 5-ALA induced fluorescence in U87MG cells incubated for 18 h with 5-ALA (Fig. S2, supplementary material). We then investigated which molecular characteristics could contribute to this difference. The U87MG cells express relatively low levels of EGFR and virtually do not express the mutant form EGFRvIII. In contrast, BS153 cells overexpress EGFR and co-express EGFRvIII, as assessed by immunofluorescence (Fig. 2b) and western blot (Fig. 2c). We further analyzed EGFR by flow cytometry, which showed a tenfold higher expression of EGFR in BS153 compared to U87MG cells. Furthermore, the expression of EGFRvIII was unique to the BS153 cell line (Fig. 2d). In LN229 overexpressing EGFR (assessed by western blot) the 5-ALA induced fluorescence was significantly higher than in BS153 (Fig. S8).

### EGF induces HO-1 expression in U87MG cells

**Fig. 3:**
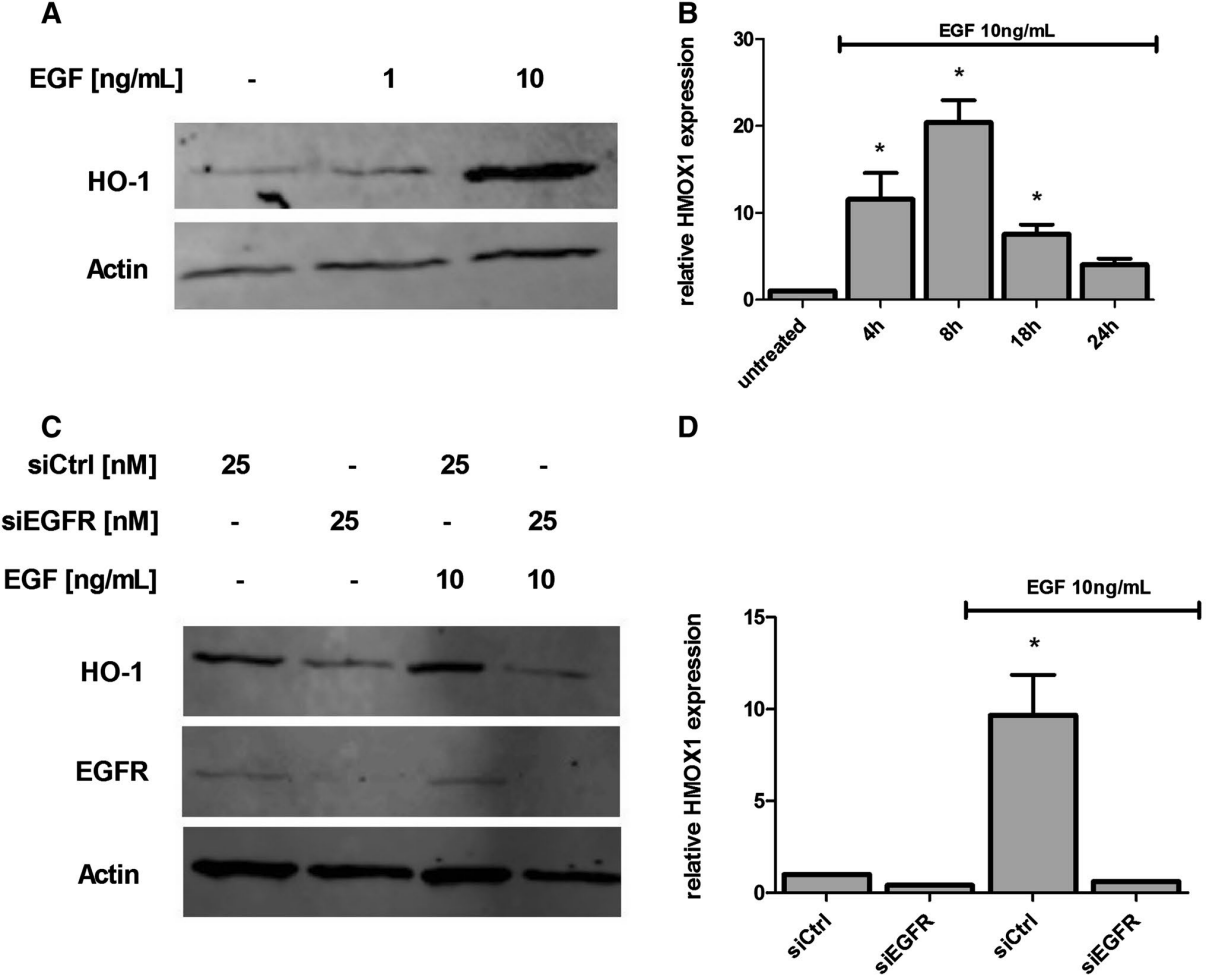
EGF stimulates HO-1 expression in U87MG cells. U87MG cells were incubated with various concentrations of EGF (1 and 10 ng/ml) for 18 h, followed by Western Blot to assess HO-1 levels (**a**). To determine *HMOX1* gene expression, cells were treated with EGF (10 ng/ml), after which total RNA was extracted. A real-time PCR was carried out as described in ‘‘Materials and methods’’ section to assess *HMOX1* mRNA levels relative to the control, untreated cells (**b**). Cells were transiently transfected with 25 nM control (siCtrl) or EGFR siRNA (siEGFR) for at least 24 h, then treated with EGF (10 ng/ml). After 18 h, cell lysates were collected and HO-1 and EGFR protein levels assessed by immunoblotting (**c**). EGFR was downregulated by siRNA-treatment in U87MG cells, followed by treatment with EGF (10 ng/ml). After 4 h, total RNA was extracted to determine *HMOX1* mRNA levels, relative to control cells transfected with transfection reagent alone (**d**). *Represents *p* < 0.05, compared to EGF treatment

We choose U87MG cells to investigate the role of the EGF signaling pathway in the regulation of HO-1 expression. Treatment with 1, 10 and 20 ng/ml EGF induced HO-1 protein expression in a concentration-dependent manner, with an approximate threefold increase at the highest concentrations when compared to untreated cells (Fig. 3a, S3). EGFR stimulation by EGF has been shown to activate NF-kB, which promotes transcription of several genes, including HO-1. Therefore, we investigated whether upregulation of HO-1 protein by EGF was occurring at the transcription level. The treatment of U87MG cells with EGF 10 ng/ml allowed us to determine that the induction of HO-1 mRNA reached its maximum at 8 h, with a 20-fold increase in transcription rate (Fig. 3b) in comparison to untreated cells. To demonstrate the involvement of EGFR in EGF-mediated induction of HO-1, we treated U87MG cells with siRNA (25 nM) specific for the EGFR. Down-regulation of EGFR strongly reduced EGF-induced HO-1 expression (Fig. 3c) and mRNA transcription (Fig. 3d) when compared to control siRNA treated cells. Therefore, our data indicate an involvement of EGFR in EGF-mediated HO-1 expression in U87MG cells.

### Selective inhibition or siRNA-mediated depletion of HO-1 increases 5-ALA fluorescence

**Fig. 4:**
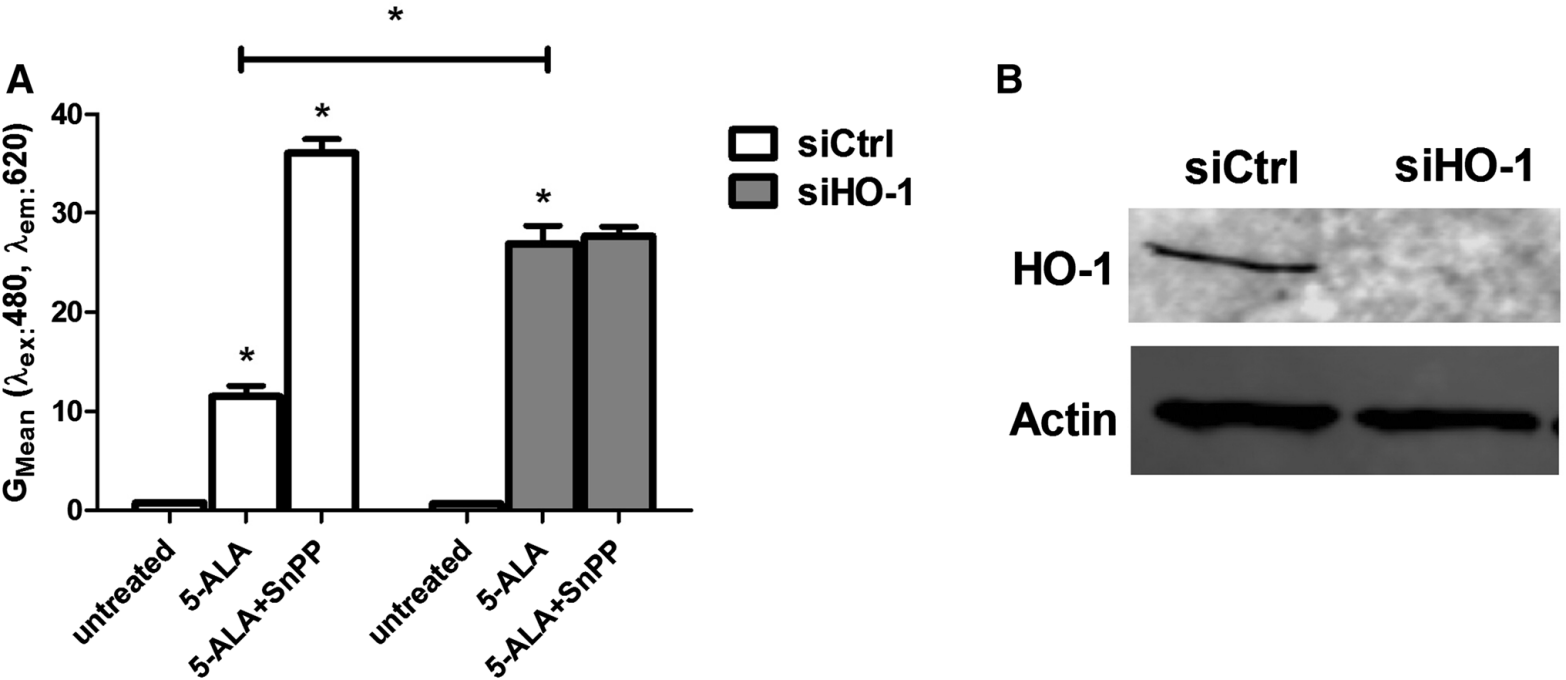
Inhibition or siRNA (siHO-1)-mediated depletion of HO-1 increases 5-ALA fluorescence in U87MG Cells. Cells were transiently transfected with either control or HO-1 siRNA, followed by incubation for 18 h with 5-ALA (1 mM) with or without the presence of the pharmacological HO-1 inhibitor SnPP (100 µM) (**a**). HO-1 protein levels of siHO-1-treated U87MG cells, determined at 48 h post-transfection (**b**). *p < 0.05 compared to untreated cells, if not specified otherwise

To test the role of HO-1 in the metabolism of PpIX, we incubated U87MG cells with SnPP, a potent inhibitor of HO-1 enzymatic activity. As described above, the presence of 5-ALA (1 mM) promoted porphyrin biosynthesis and thus cellular fluorescence. Pharmacological treatment with SnPP further increased cellular 5-ALA fluorescence (Fig. 4a, S5). In order to confirm the link between PpIX fluorescence and HO-1 expression, we down-regulated HO-1 protein expression in U87MG cells (Fig. 4b). Similarly to SnPP treatment, siRNA-mediated depletion of HO-1 enzyme significantly increased cellular 5-ALA fluorescence. The effect of siRNA-depletion was somehow inferior to that induced by SnPP. Nevertheless, treatment with SnPP in HO-1-depleted cells did not have any synergistic effect on fluorescence (Fig. 4a).

### HO-1 inhibition, reversed reduction of EGF-induced fluorescence in GBM cells independently of their EGFR/EGFRvIII quantitative expression

**Fig. 5:**
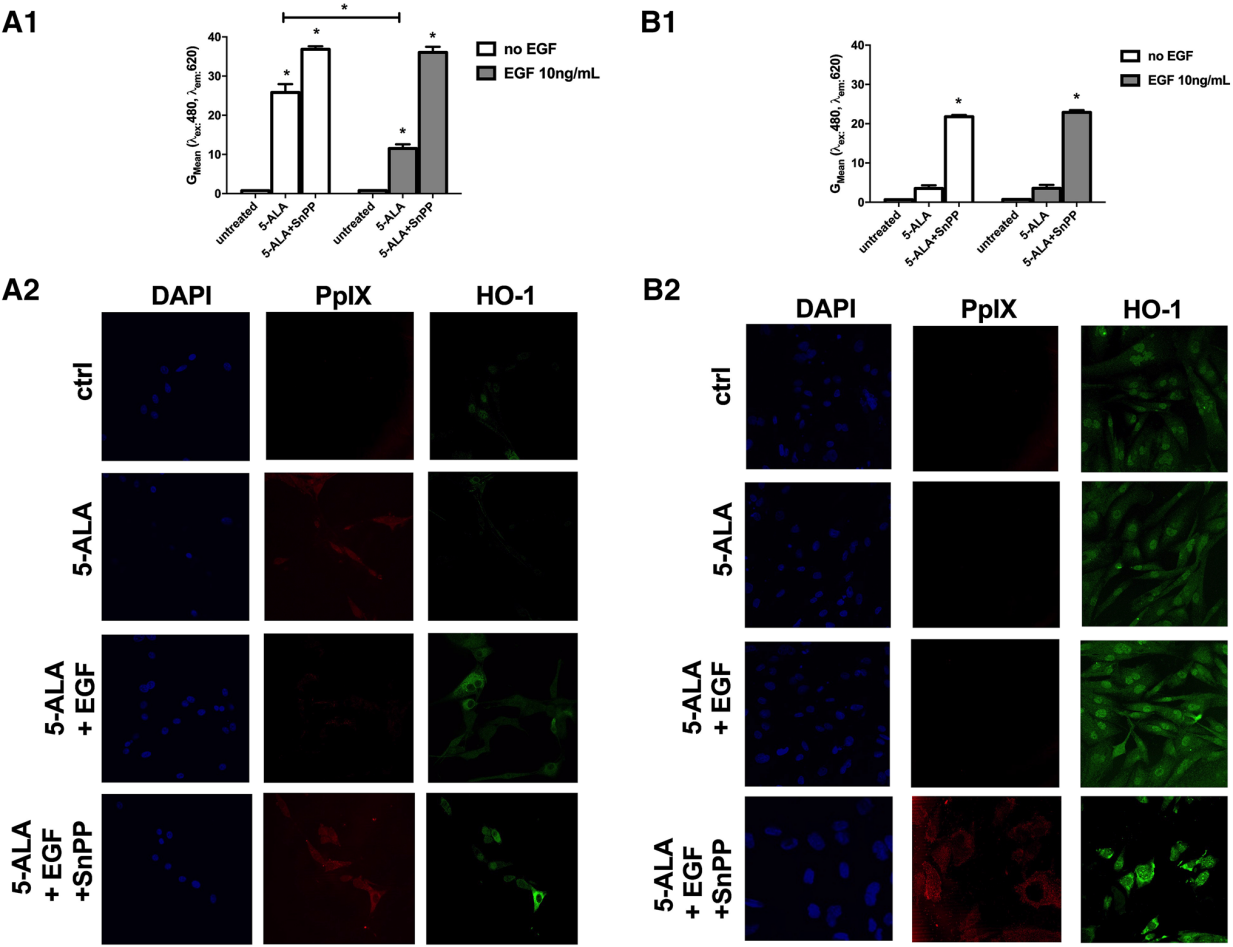
**a** EGF treatment reduces 5-ALA fluorescence in U87MG cells through induction of HO-1. U87MG cells were plated and after 24 h, medium was changed to FBS-free serum for additional 24 h. EGF (10 ng/ml) or mock treatment was added, followed by treatment with 5-ALA with or without SnPP (100 µM). Fluorescence was assessed by flow cytometry (**a**1) or by immunostaining (**a**2). *p < 0.05, compared to untreated cells, if not otherwise specified. **b** 5-ALA-induced fluorescence in BS153 cells can be restored by treatment with SnPP, a potent HO-1 inhibitor. BS153 cells were plated and, after 24 h, medium was switched to FBS-free serum for additional 24 h. EGF (10 ng/ml) or mock treatment was added, followed by treatment with 5-ALA with or without SnPP (100 µM). Fluorescence was assessed by flow cytometry (**b**1) or by immunostaining (**b**2). *p < 0.05 compared to untreated cells, if not otherwise specified

We then investigated if EGFR activation and subsequent HO-1 induction was the cause for reduced PpIX and impaired cellular fluorescence in GBM cells. In order to remove EGF from culturing conditions, cells were cultured in serum-free medium for 24 h, after which EGF (10 ng/ml) was added to the culture medium, 18 h prior to addition of 5-ALA. Consistent with the data described above, U87MG cells displayed lower 5-ALA fluorescence in the presence of EGF for 18 h when compared to EGF-depleted conditions (Fig. 5a1, a2). This effect was likely to be caused by HO-1-mediated degradation of PpIX, because selective pharmacological inhibition of HO-1 by SnPP restored PpIX fluorescence in GBM cells, independently of their quantitative expression of EGFR, as shown by Flow Cytometry (Fig. 5a1) and immunostaining (Fig. 5a2). Also at basal HO-1 expression conditions, i.e. in the absence of EGF, SnPP treatment increased PpIX fluorescence in the U87MG cells. Overall, these data confirm our hypothesis that EGF acts through its receptor to regulate 5-ALA fluorescence, and that HO-1 expression dramatically impacts 5-ALA-induced fluorescence.

### HO-1 inhibition restores 5-ALA-fluorescence in BS153 cells

Human BS153 GBM cells were used to investigate the role of EGFR/EGFRvIII expression in 5-ALA fluorescence. Analysis by flow cytometry (Fig. 5b1) and the immunostaining (Fig. 5b2) showed that inhibition of HO-1 by SnPP completely restored PpIX fluorescence in the BS153 cell line (Fig. 5b2). However, EGF-treatment had no significant effect on PpIX fluorescence in BS153 cells exposed to 5-ALA up to 5 mM in the absence or presence of SnPP (Fig. 5b2). We thus hypothesized that the lack of EGF effect and reduced PpIX fluorescence in BS153 cells was caused by the constitutive activation of the EGFR/EGFRvIII pathway, consequently inducing a constitutive over-expression of HO-1. Indeed, HO-1 mRNA levels cells were up to fivefold higher in BS153 when compared to U87MG cells (Fig. S5) and HO-1 inhibition in response to high concentration of SnPP (500 µM) restored the PpIX fluorescence in BS153 independently of the presence of EGF (Fig. 5b1 and S6). To elucidate the role of EGFR overexpression over its constitutively active form EGFRvIII, we screened LN229EGFR GBM cells (both overexpressing the EGFR protein, without expressing the constitutively active version EGFRvIII) for their 5-ALA-induced fluorescence. LN229 cell line shows high level of fluorescence as assessed by Flow Cytometry compared to BS153 (Fig. S7). Treatment with EGF 10 ng/ml could reduce fluorescence, in a proportional way with respect to the U87MG and the effect was strongly reversed by treatment with SnPP.

### Gefitinib Inhibition restored fluorescence in U87MG cells, but not in BS153 cells

Treatment with the selective Tyrosine Kinase Inhibitor (TKI) Gefitinib (10 µM) restored 5-ALA induced fluorescence of EGF stimulated U87MG cells while normalizing HO-1 activity to baseline. No effect of EGFR inhibition by Gefitinib (at 10 and 20 µM) upon 5-ALA induced fluorescence on BS153 cells was seen, maintaining constant high HO-1 activity (Fig S8).

## Discussion

Even though high grade glioma and GBM are considered diffuse and infiltrating diseases, increasing the magnitude of surgical tumor resection can impact overall survival in studies of high-grade gliomas, including GBM. 5-ALA-aided surgery has been established as an efficient tool to increase the accuracy and extent of resection of high-grade tumors and thus is part of the standard procedure during tumor removal [9]. 5-ALA induced fluorescence may vary upon many factors such as cell density, proliferation index, mitochondrial mass and, furthermore, exogenous factors such as application or fading during surgery [10, 23]. Moreover, Jaber et al. have recently shown in patients harboring gliomas, that there is a strong correlation between tumor volume, contrast enhancement, age and 18F-fluoroethyl tyrosine positron emission tomography uptake ratio, which is predictive of the fluorescence of different grade gliomas after 5-ALA administration [32]. Furthermore, during surgery, the magnitude of fluorescence may be influenced by factors such as logistical changes in time of resection after 5-ALA administration, its reabsorption, and variations in its passage through the blood brain barrier. While both 5-ALA passive and active uptake mechanisms, and their consequent implication in the time rate of the induced fluorescence, are still under discussion [5–8], the inter-observer variability of the 5-ALA-induced fluorescence remains unaddressed in the clinical setting.

It is known that PpIX, the actually fluorescent 5-ALA metabolite, has a complex biosynthesis and metabolism, with several enzymes involved. Among them, HO-1 has been shown to decrease the PpIX concentration [30]. Notably, HO-1 activity is regulated by various stimuli. Recently, EGF stimulation was shown to increase HO-1 through NF-kβ activation in colon cancer and in non-small cell lung cancer [26, 33].

In the present study, we investigated the role of EGF in the metabolism of 5-ALA as a possible cause of variability in tumor-associated fluorescence in GBM cells. In our human cell tumor cultures, EGF stimulation induced a significant increase in HO-1, both at mRNA and protein expression levels, and a significant reduction in 5-ALA fluorescence. The data indicate that these effects were directly correlated with activation of the EGFR, as siRNA mediated knockdown of EGFR significantly hampered EGF-dependent HO-1 induction.

PpIX is not a direct substrate for HO-1 enzymatic activity. Instead, it must be converted into heme by the enzyme FECH before it can be further metabolized by HO-1. We hypothesized that the EGF-mediated increase in HO-1 would result in accelerated heme depletion and thus a shift in enzymatic activity in favor of increased PpIX metabolism by FECH. Supporting this notion, we show that pharmacological blockade of HO-1 by SnPP significantly increased cellular 5-ALA fluorescence. Similarly, siRNA mediated down-regulation of HO-1 increased cellular fluorescence whilst abolishing the SnPP effect. Taken together, our data show that increased EGFR activity results in up-regulation of HO-1-mediated heme clearance and, ultimately reduced PpIX fluorescence.

BS153 cells overexpress EGFR as well as the constitutively active EGFRvIII+. We thus assumed that even in the absence of any EGF stimulation, BS153 cells would display increased levels of HO-1 protein and reduced PpIX fluorescence. Indeed, even in EGF-depleted, serum-free culturing conditions, no fluorescence was observed in BS153 cells. Treatment with high-dose SnPP (up to fivefold compared to U87MG cells) was able to restore PpIX fluorescence in BS153 cells, clearly highlighting the role of HO-1 in 5-ALA-induced, or lack of, GBM tumor fluorescence. Interestingly LN229EGFR cells, which do overexpress EGFR but are negative for EGFRvIII, show high levels of 5-ALA-induced fluorescence, comparable with U87MG cells. The observation that 5-ALA fluorescence is dependent on the activity of HO-1, which in turn is dependent of the activity of EGFR, is clinically relevant as it underlies the clinical findings of possible low or absence of fluorescence to the naked eye as reported by Hauser et al. Thus, together our findings suggest that intra-tumoral heterogeneity of EGFR/EGFRvIII expression might explain the intra-tumoral variability in 5-ALA induced fluorescence expression, resulting, as previously reported, in false negative areas of tumor infiltration [10, 23].

Interestingly Gefitinib, a selective inhibitor of EGF tyrosine kinase receptor, was not capable of restoring PpIX fluorescence in BS153 cell cultures. Levels of Gefitinib sufficient to suppress EGFRwt phosphorylation (0.01–0.1 µM), have been previously shown to be insufficient to inhibit the same feature in cells expressing EGFRvIII [34], suggesting the difficulty of blocking the mutated and constitutively active EGFR receptor. Our results further support those findings, as a concentration of Gefitinib approximately tenfold higher than that used to inhibit EGFRvIII in different cell lines was not enough to restore the PpIX fluorescence in BS153 cells. Together, the data suggests that the expression level of EGFRvIII and its varying degree of resistance to inhibition by Gefitinib in the clinical setting, may justify why GBM tumor tissue can be present yet not visible by 5-ALA-induced fluorescence.

Taken together, our findings seem to indicate that EGFR plays a relevant role in cellular fluorescence upon treatment with 5-ALA. Notably, the presence of EGFRvIII, which is constitutively active even in absence of EGF, strongly hampers fluorescence. Overexpression of EGFR alone seems not to influence the intensity of fluorescence; nevertheless upon treatment with EGF, fluorescence was strongly reduced in these cells, highlighting the role of increased EGF-induced HO-1 expression and turn-over of PpIX.

These findings do not disagree with the utility of fluorescence assisted resection surgery; instead the data points to a problem strongly impacting the definition of the tumor border, and draws a possible justification for the cases of reported low or no visible 5-ALA induced fluorescence despite histological confirmation of the tumor presence, especially at the infiltrating zone. These findings highlight the need to evaluate clinical databanks, in order to confirm or negate the evidence suggested by the in vitro results. However, our in vitro results support an explanation for the event of low, or absence of, visible 5-ALA induced fluorescence in histologically-confirmed GBM, which contributes for a new perspective of the problem and inviting to rethink a strategy to address these questions.

## Electronic supplementary material

Below is the link to the electronic supplementary material.

Supplementary material 1 (JPG 42 KB)

Supplementary material 2 (JPG 170 KB)

Supplementary material 3 (JPG 376 KB)

Supplementary material 4 (JPG 288 KB)

Supplementary material 5 (JPG 104 KB)

Supplementary material 6 (JPG 301 KB)

Supplementary material 7 (JPG 1495 KB)
