# Supplementary figures and images for: Epithelial growth factor receptor expression influences 5-ALA induced glioblastoma fluorescence
Source: J Neurooncol. 2017 May 12;133(3):497–507. doi: 10.1007/s11060-017-2474-0 (PMC5537329; doi:10.1007/s11060-017-2474-0)

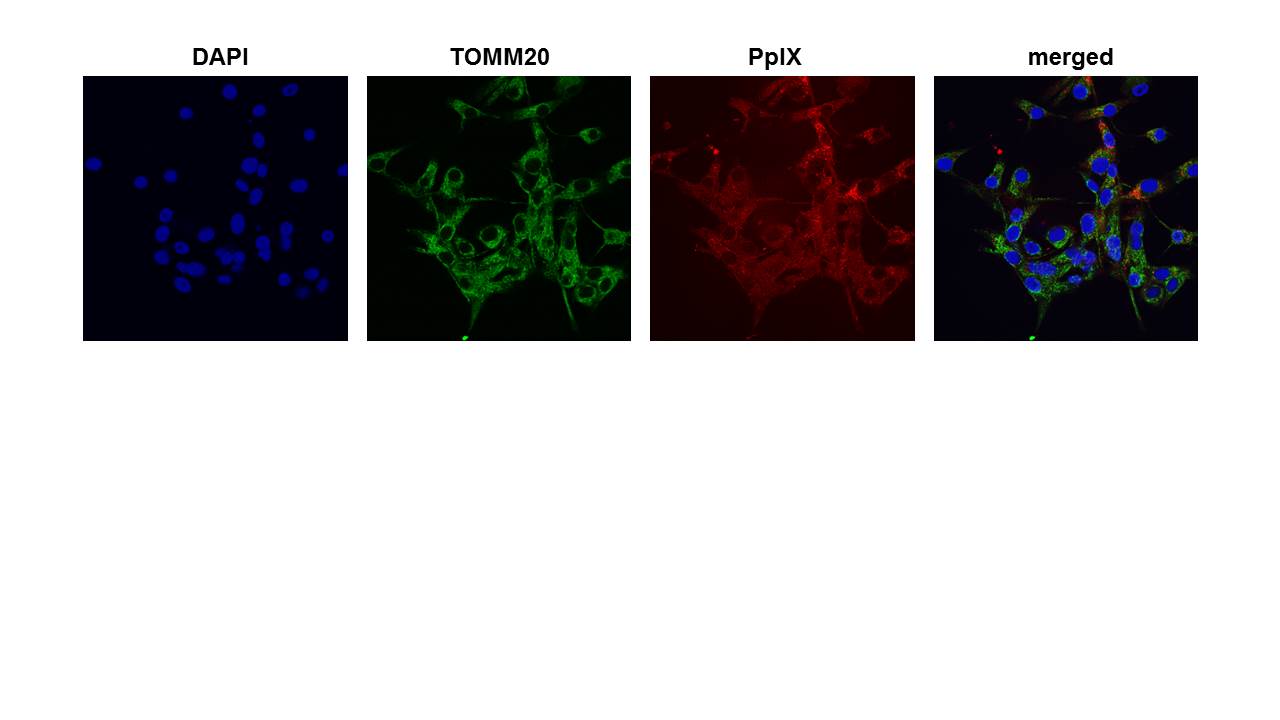

Supplement: Supplementary file 1 — Supplementary material 1 (JPG 42 KB) [file 11060_2017_2474_MOESM1_ESM.jpg]

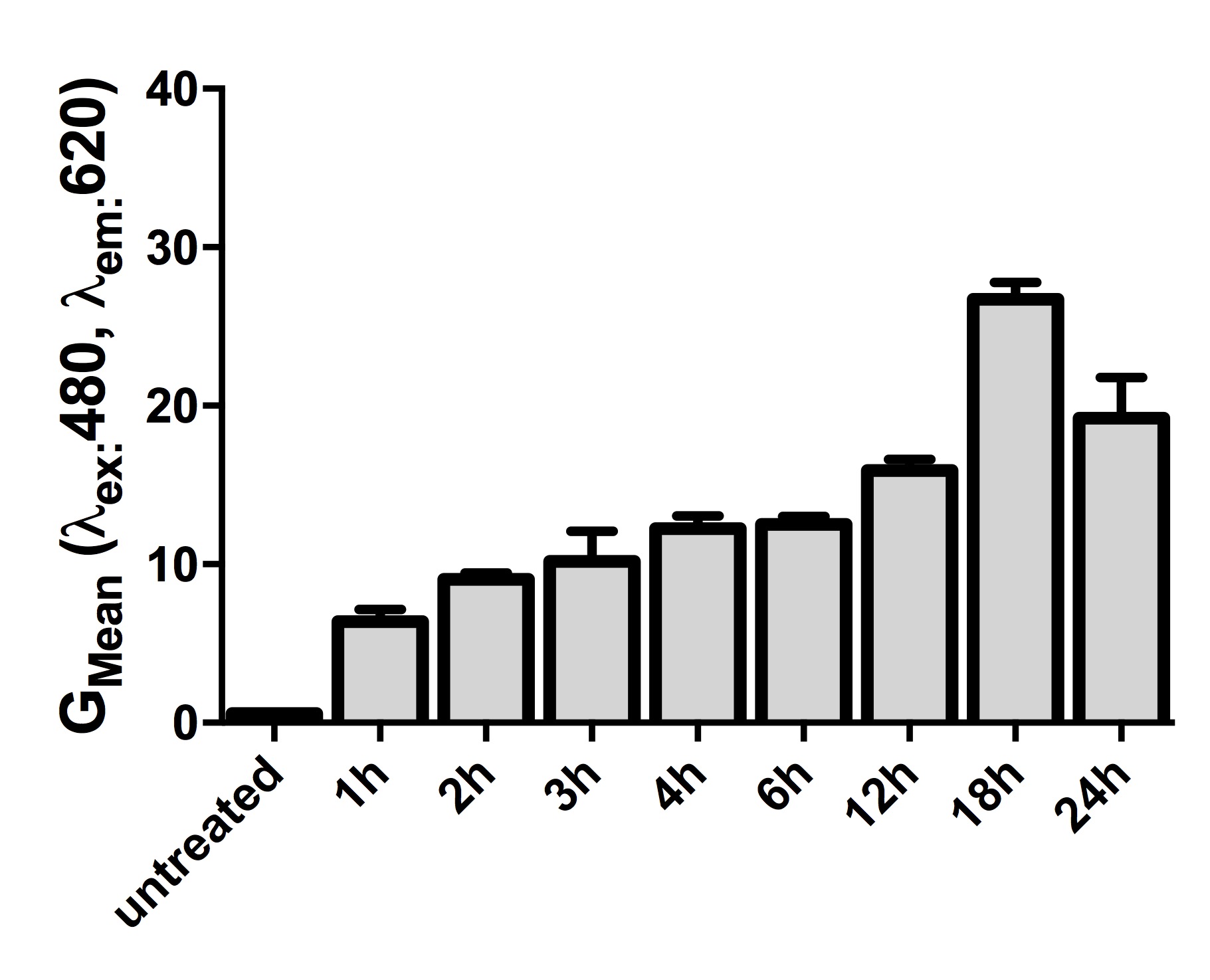

Supplement: Supplementary file 2 — Supplementary material 2 (JPG 170 KB) [file 11060_2017_2474_MOESM2_ESM.jpg]

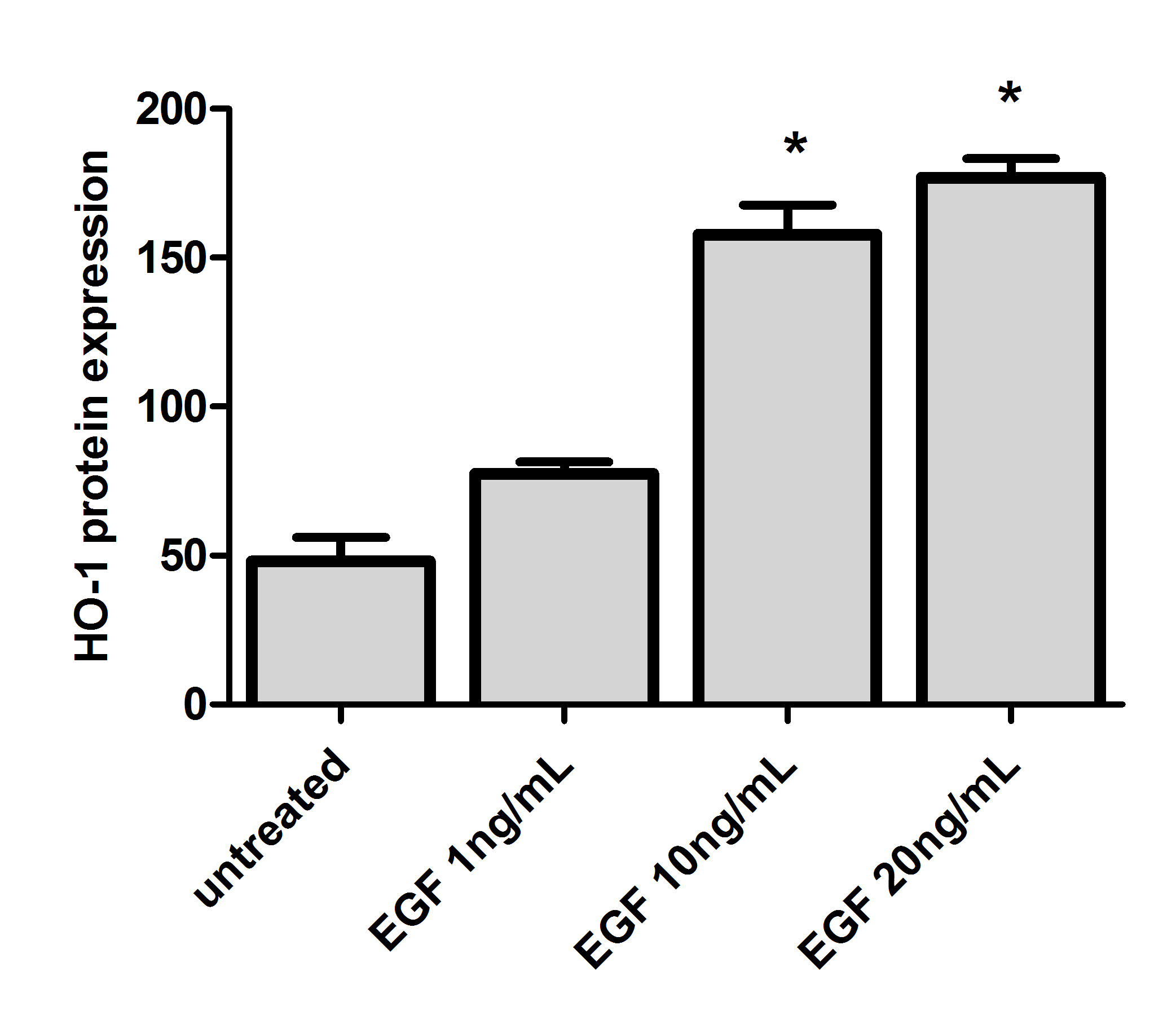

Supplement: Supplementary file 3 — Supplementary material 3 (JPG 376 KB) [file 11060_2017_2474_MOESM3_ESM.jpg]

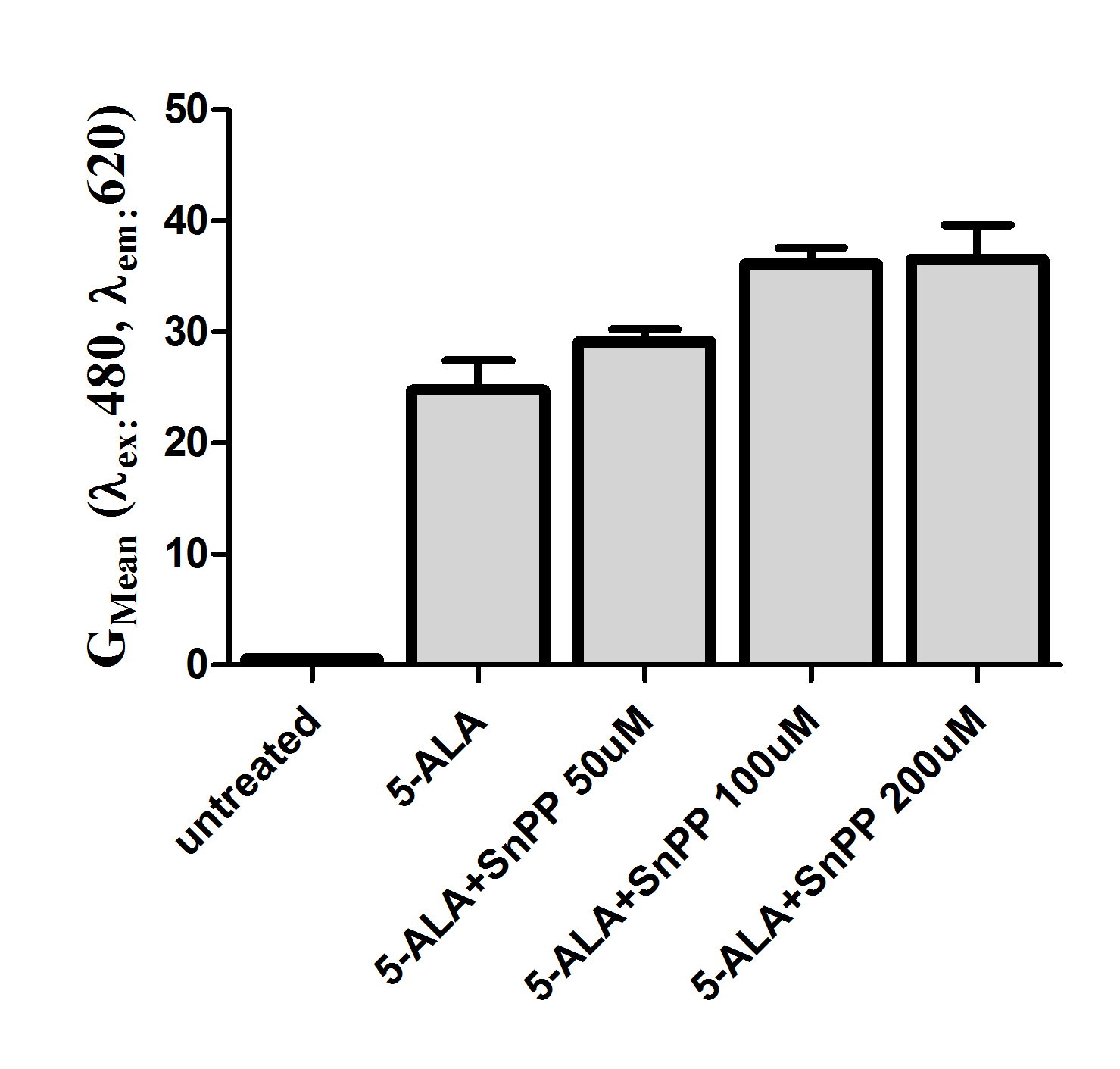

Supplement: Supplementary file 4 — Supplementary material 4 (JPG 288 KB) [file 11060_2017_2474_MOESM4_ESM.jpg]

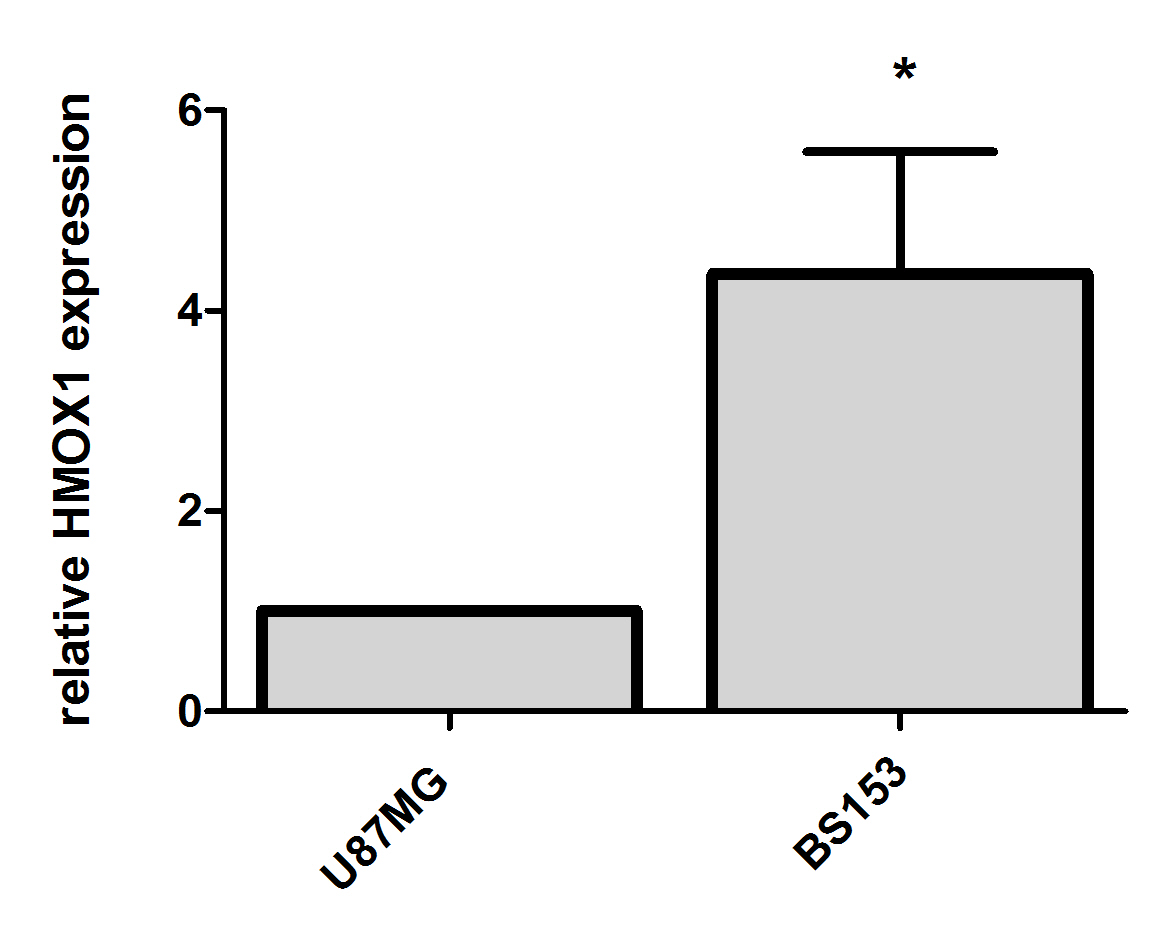

Supplement: Supplementary file 5 — Supplementary material 5 (JPG 104 KB) [file 11060_2017_2474_MOESM5_ESM.jpg]

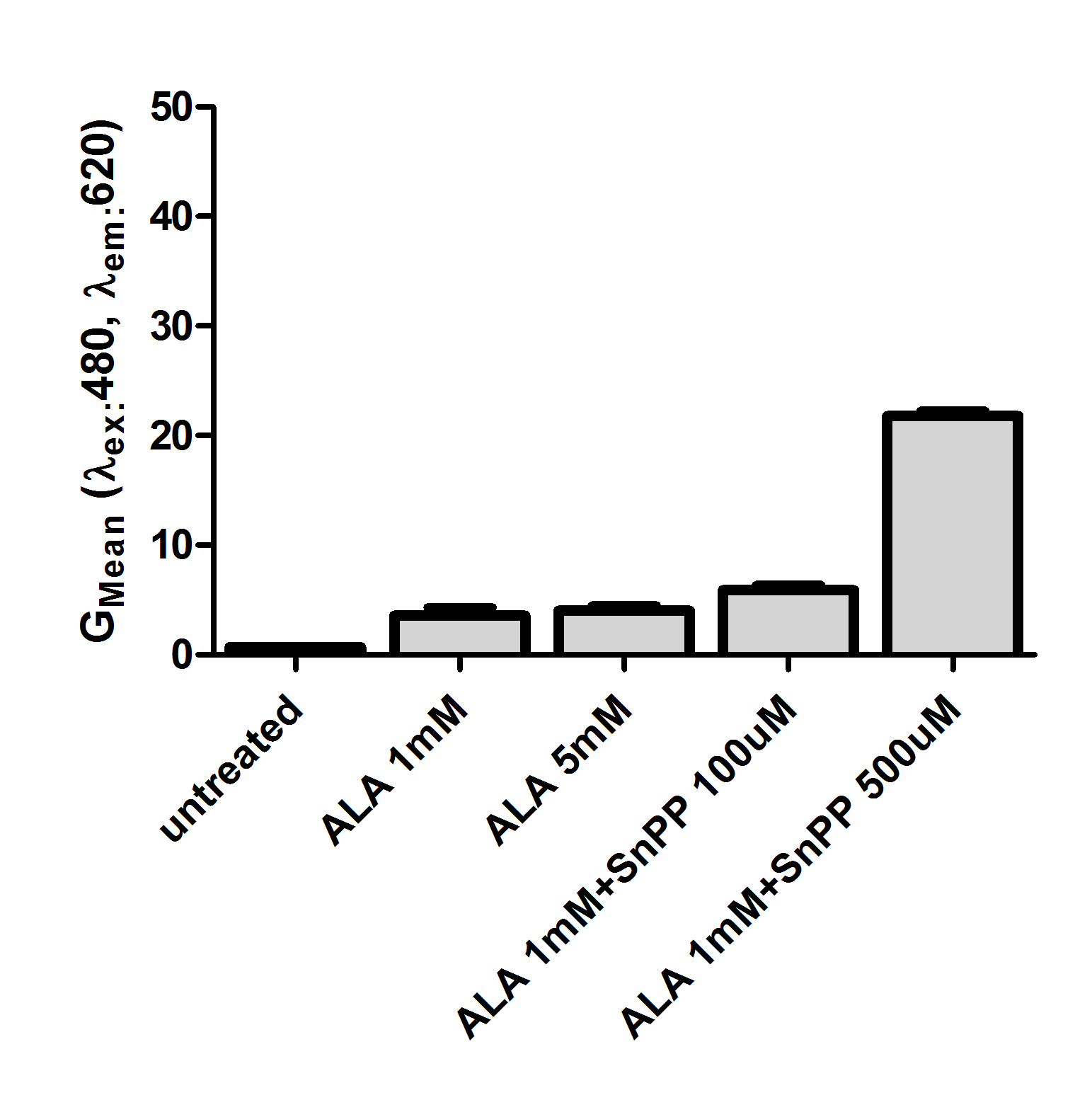

Supplement: Supplementary file 6 — Supplementary material 6 (JPG 301 KB) [file 11060_2017_2474_MOESM6_ESM.jpg]

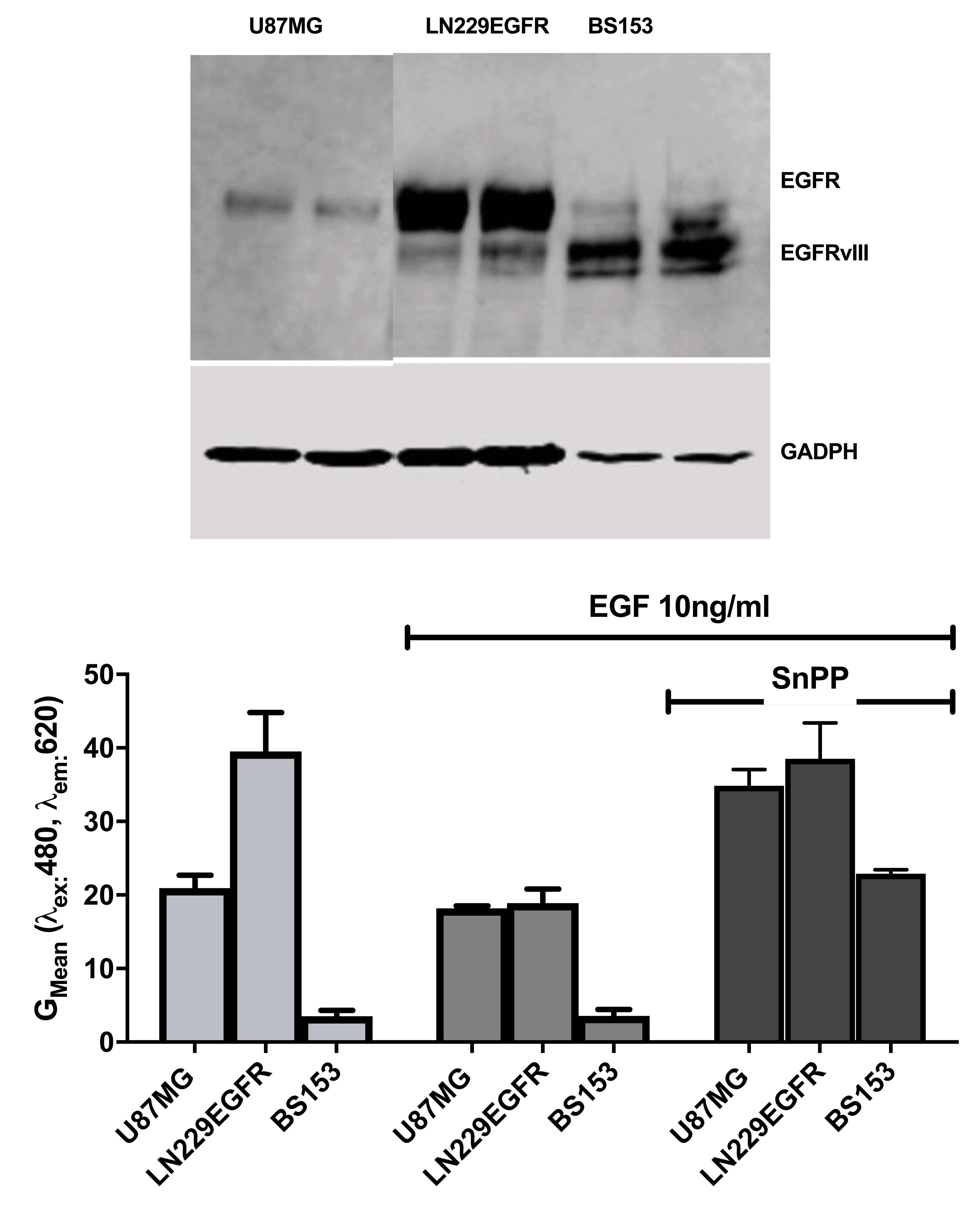

Supplement: Supplementary file 7 — Supplementary material 7 (JPG 1495 KB) [file 11060_2017_2474_MOESM7_ESM.jpg]
